# Quantification of spinal ataxia in dogs with thoracolumbar spinal cord injury

**DOI:** 10.3389/fvets.2023.1183755

**Published:** 2023-08-08

**Authors:** Tamara Sherif, Friederike Twele, Sebastian Meller, Alexandra Müller-Anders, Holger A. Volk

**Affiliations:** Department of Small Animal Medicine and Surgery, University of Veterinary Medicine Hannover, Hannover, Germany

**Keywords:** spinal cord injury, ataxia, dog, gait analysis, locomotion, movement

## Abstract

The clinical sign of ataxia is related to several neurological diseases and is seen in conjunction with paresis in dogs with spinal cord injury (SCI). Endeavours to objectify canine spinal ataxia in SCI remain limited. The aim of this clinical study was to determine and quantify differences between gait characteristics of ataxic dogs with thoracolumbar myelopathy and healthy control dogs using a computer-and treadmill-based gait analysis system. Five dogs with spinal ataxia and six healthy dogs underwent video-and computer-assisted gait analysis while walking on a four-ground reaction force plate treadmill system (maximum speed of 0.7 m/s). Spatio-temporal and kinetic gait characteristics regarding the dogs’ locomotion were analysed with a focus on the individual coefficient of variation (CV), as a potential measure for quantification of the level of ataxia. Ataxic dogs with thoracolumbar SCI showed no effect on symmetry indices but higher variability in spatio-temporal and kinetic gait parameters mainly in the pelvic, but also in the thoracic limbs. Double support phase of the individual limb was prolonged in SCI dogs at the cost of the single support and swing phase. Reduced peaks of ground reaction forces (GRF) could potentially be explained by reduction of muscle strength, as a strategy of avoiding falling by taking enthusiastic steps, or by alteration of the rhythmogenic spinal circuits between the pelvic and thoracic limb pattern generators.

## 1. Introduction

The neurological clinical sign of ataxia is defined as disturbed coordination of movement and can affect interaction between muscle groups (1) and adjustment of body axis orientation (2). Incoordination can be considered as the inability to maintain a cyclical interaction of temporal and spatial gait parameters, especially between the extremities and the body center (3). However, ataxia is most frequently observed in dogs with spinal cord injury (SCI) (4), secondary to intervertebral disc disease (IVDD) (5) and is then likely associated with paresis (1).

Prognostic factors do not only include severity of initial injury and its accompanying severity of neurological deficits, but also treatment options available and time until recovery of ambulation (6). Moreover, quality of life is often impacted in these patients (7). Further studies on these topics require individualized and objective spinal ataxia data, which can help study SCI in more granularity.

Interobserver reliability of observational gait analysis has been widely studied and shown to be dependable, provided that the analysis parameters are clearly defined and limited to a manageable quantity for the observer. Although observational gait analysis is an effective tool for clinical reasoning, the human eye, even in slow motion, is unable to fully analyse the complex components of the biped’s locomotion taking place within milliseconds, regardless of the observer’s degree of experience (8–10). Therefore, objective measures, such as those generated on force plate treadmills, can complement ordinal gait scales and precisely reveal different aspects in detail.

Although gait aberrations caused by neurologic dysfunctions have been a well-researched topic (11–22) there is still evidence missing on the benefits of a four-belt force plate treadmill system regarding its potential to objectively quantify ataxia, gait and step parameters, their coefficients of variation (CVs) and the relationship between the individual limbs. Four-belt force plate treadmill gait analysis is considered gold standard in veterinary orthopaedics, particularly because of their sensitivity with regard to kinetic parameters. To the best of authors’ knowledge this is the first four-belt force plate treadmill-based study with a focus on CVs of spatio-temporal and kinetic gait parameters in ambulatory dogs with SCI. The aim of the current study was to evaluate if the variability of temporal and spatial gait characteristics, as well as ground reaction forces (GRF, kinetics) of dogs with SCI-induced ataxia detected by a four-belt force plate treadmill system differ significantly from those of a healthy control group. GRF is the force exerted between a body and the ground when in contact with it. In a standing position, this force corresponds to the weight of the body, but it changes when the body is in motion. If differences to healthy dogs can be detected, the gait analysis system could provide a basis for further studies and help in the quantification of ataxia to monitor the recovery of patients in treatment trials.

## 2. Methods

Data collection was performed according to the German Law on Animal Protection and the EU Council Directive 210/63/EU. An ethical committee officially granted ethical approval for this study (reference number for this project: 20A555) based on §15 of the German animal welfare act together with the government agency of the Lower Saxony State Office for Consumer Protection and Food Safety (LAVES). LAVES regulations limited the number of participants based on a previously conducted power analysis (G*Power, HHU University of Duesseldorf) after taking into consideration studies on similar populations and parameters.

### 2.1. Data collection

**Figure 1:**
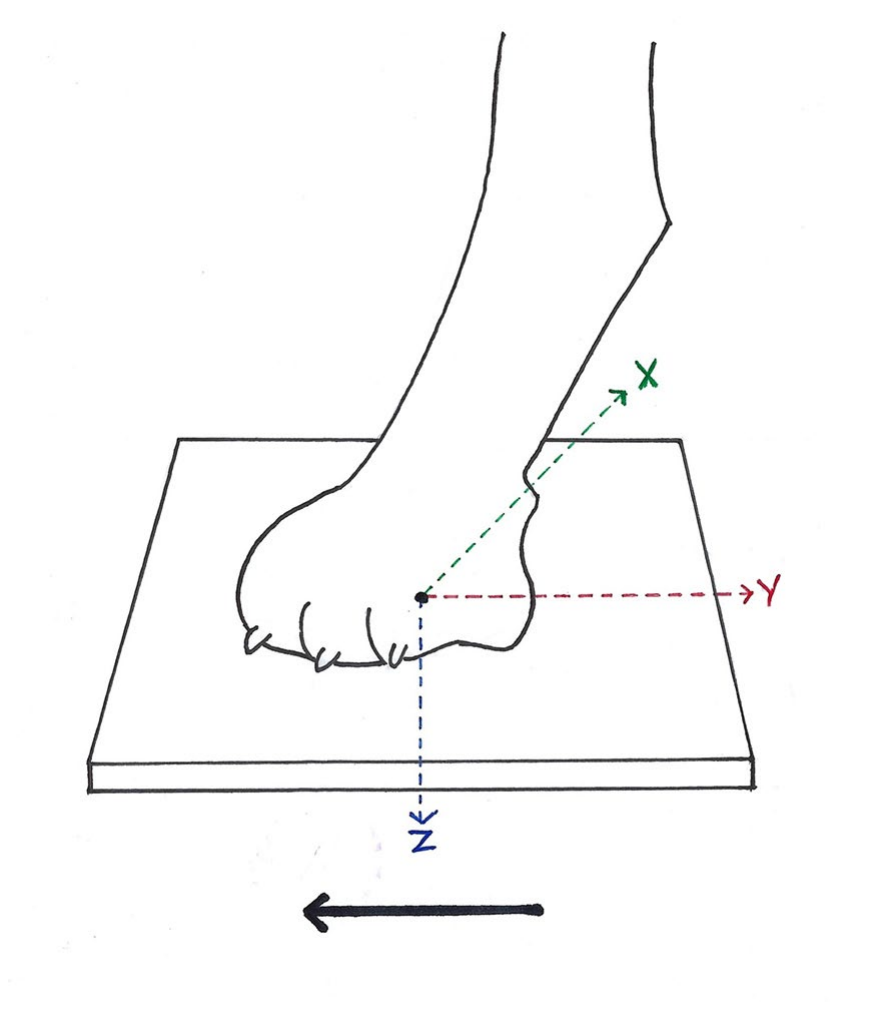
Ground reaction forces (GRF) measurable during stance phase of each paw. While walking in the direction of the solid black arrow, each paw was individually placed on one belt of the four-belt treadmill. GRF in vertical direction (Fz, blue arrow), GRF in cranio-caudal direction (Fy, red arrow) and GRF in medio-lateral direction (Fx, green arrow) were measured, according to McLaughlin et al. (28). Solid black arrow = direction of forward progression.

Data collection was performed at the Department of Small Animal Medicine and Surgery of the University of Veterinary Medicine Hannover similar to previous studies (23–26), especially to one study on ataxic dogs receiving phenobarbital (11). In this prospective clinical study, gait parameters of clinic-owned beagle dogs (control group) were compared to a study group of patients with thoracolumbar SCI. In this study neurologically caused incoordinations were examined. Dogs with a history of an orthopaedic condition or an abnormal orthopaedic examination were excluded from the study. Furthermore, in order to exclude presence of general diseases that were incompatible with participating in this study (i.e., cardiovascular insufficiency or hyperaesthesia), general examination was performed in advance. All dogs had a neurological and orthopaedic examination and only dogs with a T3/L3 myelopathy, further characterised by magnetic resonance imaging (MRI), were included. Kinetic gait parameters were collected as established and formerly described for the gait lab at the University of Veterinary Medicine Hannover (11, 23–26). Kinetic analysis provides limb-specific information and is defined as interpretation of forces occurring during motion of subjects or objects particularly in regards of ground reaction forces transmitted to force plates by the dog’s extremities. Kinetics are established as a useful tool in objective evaluation of musculoskeletal conditions as well as different therapeutic options (27, 28). The gait laboratory at the University of Veterinary Medicine Hannover contains three components: a treadmill with four integrated force measuring plates, a three-dimensional gait analysis system including high speed cameras as well as gait analysis software (29). It enables the observer to objectively measure ground reaction forces (Figure 1). The electronic four-belt treadmill (TM-07-B, Bertec Corp, United States) for detection of locomotion of participating dogs was complemented by a digital high-speed video camera (pilot piA640-210gc; Basler, Germany), videotaping from the left body position of the walking dog. All devices listed were monitored by Bertec Treadmill Control Panel software (ver 1.7.12; Bertec Corp, United States), as well as Vicon Nexus software (ver 1.8.5; Vicon Motion Systems Ltd., United Kingdom). Motion capture system was calibrated prior to use to adapt it to current conditions and an adaptation phase prior to recording was needed until the dogs were able to walk in a natural manner. For calibration a three-dimensional T-shaped calibration stick with reflective markers attached was manually moved in a random pattern above the area recorded for analysis until the video-system recognized all markers in all angles of the three-dimensional space and until the force plate measure was nulled when not carrying any weight. With this procedure the treadmill-based gait analysis system tests its functionality, inhibits incorrect measurements and reduces artifacts. In order to enable a smooth and regular walking session, treadmill speed was adjusted to 0.7 m/s.

### 2.2. Data processing

For each participating dog, recorded gaits were reviewed to eliminate videos where gait might have been biased by turning of the head during walking or walking speed changes for further analysis of 50 complete stride cycles. If 50 complete stride cycles could not be measured in one trial run, multiple fragments of at least 10 continuous stride cycles each of adequate quality were used for further analysis. The number and length of trials and breaks in between trials was individually adapted to generate sufficient stride cycles without artifacts. Data of GRF were filtered through a low-pass 10-Hz finite-impulse response filter. The events “strike-down” and “lift-off” of paws were implemented by being linked to the high-definition video material as well as start and end of detection of vertical ground reaction forces during one stride. Captured data sets were exported to a Microsoft Excel 2010 spreadsheet (Microsoft Corp, United States). Table 1 shows definitions of spatio-temporal and kinetic gait parameters measured or calculated on behalf of this study.

**Table 1:** Definitions of gait parameters measured or calculated on behalf of this study.

Parameter	Unit	Definition	Further information
**Spatio-temporal**			
Stride length	mm	Distance between paw marker position of ipsilateral limb during one stride	Treadmill belt length that passed during full gait cycle
Stride time	s	Time between successive ipsilateral paw strikes	Full gait cycle
Step length	mm	Distance between paw marker position of ipsilateral limb during one step	
Step time	s	Time between contralateral and successive ipsilateral paw strikes	
Rel Step length	%	Step length/stride length*100	
Rel Step time	%	Step time/stride time*100	
Stance time	s	Time between ipsilateral paw strike and successive paw off	
Swing time	s	Time between ipsilateral paw off and successive paw strike	
Single support phase	%	Time from contralateral paw off to successive contralateral paw strike/stride time*100	Single support phase + double support phase + swing phase = 100% stride
Double support phase	%	(Time from ipsilateral paw strike to contralateral paw off + time from contralateral paw strike to ipsilateral paw off)/stride time*100	Single support phase + double support phase + swing phase = 100% stride
Swing phase	%	Time between ipsilateral paw off and successive paw strike/stride time*100	Single support phase + double support phase + swing phase = 100% stride
**Kinetic**			
PFz	%	Maximum (peak) vertical ground reaction force during one stride in relation to body weight	
MFz	%	Mean vertical ground reaction force during one stride in relation to body weight	
IFz	New*s/%	Impulse of vertical ground reaction forces during one stride in relation to body weight (MFz*stance time)	
Range Fz	New	Mean range of vertical ground reaction forces during one stride	
STDEV Fz	New	Standard deviation of vertical ground reaction forces during one stride	
Relation PFz T/P	-	Distribution of vertical ground reaction force comparing thoracic to pelvic limbs (PFz thoracic limb/PFz pelvic limb)	Common: 60/40–70/30 = 1.5–2.3
Symmetry index PFz	%	SI = 100% − [(PFz left paw /PFz right paw)*100] [modified formula by Budsberg et al. (30)]	>6/<−6 = orthopedic lameness
PFy	%	Maximum (peak) craniocaudal ground reaction force during one stride in relation to body weight	
Range Fy	New	Mean range of craniocaudal ground reaction forces during one stride	
STDEV Fy	New	Standard deviation of craniocaudal ground reaction forces during one stride	
PFx	%	Maximum (peak) mediolateral ground reaction force during one stride in relation to body weight	
Range Fx	New	Mean range of mediolateral ground reaction forces during one stride	
STDEV Fx	New	Standard deviation of mediolateral ground reaction forces during one stride	

T, thoracic limb; P, pelvic limb; Walking speed, stride length/stride time.

All GRF data were standardized for each participating dog by expressing data as percentage of its’ body weight using the following equation:GRF = Fz; Fy; Fx/(body weight*9.81); (9.81 = gravitational force in new/kg).

Moreover, symmetry indices, relative step length and relative step time were estimated using the following equations:

Symmetry index (SI) = 100% − [(PFz left paw/PFz right paw)*100] [modified formula by Budsberg et al. (30)].

Relative step time = step time/stride time.

Relative step length = step length/stride length.

Finally, CV of all gait parameters recorded were calculated, as used in multiple previous canine ataxia studies (12, 13, 31), horses (32) or humans (33) to enable relative comparison:

CV = Standard deviation/mean.

### 2.3. Statistical analysis

Statistical testing was conducted by GraphPad Prism 9 software (GraphPad Prism 9.2.0, GraphPad Software, Inc., United States). Differences in age, sex status and body weight between the groups were tested via unpaired *t*-test. Values of gait parameters and CVs were inspected for Gaussian distribution via Kolmogorov–Smirnov test and subsequently residual plots were visually estimated. After normality of distribution was rejected absolute parameter values of participating groups were statistically compared using the multiple Mann–Whitney test. *p*-values of less than 0.05 were considered as significant outcome for all statistical tests. *p*-values were then reviewed using a False Discovery Rate (FDR) in order to reduce Type-I error of multiple comparisons. The method selected for setting FDR was designed by Benjamini et al. (34). Data are shown as median with range in case of rejection of normal distribution and as mean with standard deviation if normal distributed.

## 3. Results

### 3.1. Study participants/animals

Eleven dogs of different ages, sex, breeds and body weights participated in this study. The control group consisted of 6 healthy Beagle dogs (age 4 [2–4] years; body weight 15.5 [12.7–16.7] kg). On the day of gait analysis, the control group needed an adaptation phase to the treadmill of 8 ± 4.56 min. The SCI group consisted of 5 dogs (1 Australian Shepherd, 1 Beagle, 2 Dachshunds, 1 Podenco; age 6 [5–7] years; body weight 9.9 [8.3–16.2] kg) that showed no signs of general nor orthopaedic disease but ataxia as a clinical sign of thoracolumbar myelopathy. SCI in all dogs of the study group was chronic, confirmed via MRI. All dogs were able to stand and take at least ten consecutive stride cycles without any external support and thus were considered ambulatory. All patients’ owners provided written informed consent for their dog to participate in the study. Four of the dogs had an intervertebral disc extrusion and underwent a hemilaminectomy for surgical decompression and one dog’s MRI revealed multiple degenerative compressive lesions in the thoracolumbar spinal cord. The owner elected against a surgical decompression. On the day of gait analysis, the study group required an adaptation phase to the treadmill of 22.4 ± 7.96 min. After individual adaptation, study participants of both groups were able to place their feet on each treadmill belt without any major difficulties. Thus, the amount of video material not usable for consecutive analysis was negligible. There was no statistical difference between sex status (*p* = 0.3527) and body weights (*p* = 0.0896) between the control group and the group of dogs with SCI. However, there was a difference in age between the groups with the control group being younger (*p* = 0.0007).

### 3.2. Spatio-temporal gait parameters

#### 3.2.1. Parameter values of spatio-temporal gait parameters

Comparing parameter values of 50 strides in Table 2 showed significant differences between the groups regarding stride length, step length, stride time, step time, single support phase, double support phase and swing phase of the thoracic and pelvic limbs as well as stance time of the thoracic limbs.

**Table 2:** Statistical analysis of parameter values and coefficients of variation (*p ≤ 0.05, Mann–Whitney test, FDR Q = 0.05) of spatio-temporal gait parameters.

SPATIO-TEMPORAL	Parameter values		Coefficients of variation	
	*p* value	FDR threshold	*p* value	FDR threshold
Stride length T	<0.0001*	0.0042	0.0238*	0.0240
Stride length P	<0.0001*	0.0050	0.0476*	0.05
Step length T	<0.0001*	0.0060	0.0238	0.05
Step length P	<0.0001*	0.0074	0.0238*	0.0375
Stride time T	<0.0001*	0.0094	0.0173	0.0122
Stride time P	<0.0001*	0.0122	0.0087	0.0074
Step time T	<0.0001*	0.0167	0.0519	0.05
Step time P	<0.0001*	0.0240	0.0043	0.0042
Stance time T	<0.0001*	0.0375	0.0043*	0.0050
Stance time P	<0.0001	0.05	0.0087*	0.0094
Swing time T	<0.0001	0.05	0.0173	0.0167
Swing time P	0.4235	0.05	0.0043*	0.0060
rel. Step length T	0.0320	0.0313	0.0952	0.05
rel. Step length P	0.2208	0.05	0.0238*	0.0313
rel. Step time T	0.7065	0.05	0.1808	0.05
rel. Step time P	0.6766	0.05	0.0090*	0.0200
Single support T	<0.0001*	0.0050	0.0087*	0.0139
Single support P	<0.0001*	0.0062	0.0043*	0.0050
Double support T	<0.0001*	0.0078	0.0519	0.05
Double support P	<0.0001*	0.0102	0.0043*	0.0062
Swing phase T	<0.0001*	0.0139	0.0043*	0.0078
Swing phase P	<0.0001*	0.0200	0.0043*	0.0102

Fifty strides of a non-ataxic control group as well as 50 strides of an ataxic study group were analyzed and compared. T, thoracic limbs; P, pelvic limbs; *N*, number of steps; CV, coefficient of variation; Highlighted cells, absolute values (restricted comparability); see Table 1 for detailed definitions of spatio-temporal and kinetic gait parameters and Supplementary Table S1 for descriptive statistics.

#### 3.2.2. Coefficients of variation of spatio-temporal gait parameters

Table 2 shows that between controls and the group of dogs with spinal ataxia, differences were seen for CVs of stride length, stance time, single support phase and swing phase of both limb pairs. Furthermore, CVs of step length, relative step length, relative step time, swing time and double support phase of the pelvic limbs differed between groups. In all cases, dogs with SCI showed higher variability.

### 3.3. Kinetic gait parameters

#### 3.3.1. Parameter values of kinetic gait parameters

As presented in Table 3, differences were detected between the groups concerning PFz, IFz, PFy, as well as range and standard deviation (STDEV) of Fz and Fy of both limb pairs. Besides, comparison of the parameters MFz and range of Fx of the thoracic limbs revealed significant differences. No significant differences between the groups concerning other kinetic parameter values including symmetry indices (Supplementary Table S3) were found.

**Table 3:** Statistical analysis of parameter values and coefficients of variation (^*^p ≤ 0.05, Mann–Whitney test, FDR *Q* = 0.05) of kinetic gait parameters.

KINETIC	Parameter values		Coefficients of variation	
	*p* value	FDR threshold	*p* value	FDR threshold
PFz T	<0.0001*	0.0042	0.0823	0.05
PFz P	<0.0001*	0.0050	0.0173	0.0102
MFz T	<0.0001*	0.0060	0.0173	0.0139
MFz P	0.1907	0.05	0.0043*	0.0050
PFy T	<0.0001*	0.0074	0.0043*	0.0062
PFy P	<0.0001*	0.0094	0.2468	0.05
PFx T	0.1019	0.0375	0.6623	0.05
PFx P	0.5795	0.05	0.0173*	0.0200
IFz T	<0.0001*	0.0122	0.0173*	0.0313
IFz P	<0.0001*	0.0167	0.0043*	0.0078
Range Fz T	<0.0001*	0.0042	0.1255	0.05
Range Fz P	<0.0001*	0.0050	0.0823	0.0167
STDEV Fz T	<0.0001*	0.0060	0.1255	0.05
STDEV Fz P	<0.0001*	0.0074	0.0519	0.0060
Range Fy T	<0.0001*	0.0094	0.0519	0.0074
Range Fy P	<0.0001*	0.0122	0.0173	0.0050
STDEV Fy T	<0.0001*	0.0167	0.0087	0.0042
STDEV Fy P	<0.0001*	0.0240	0.0519	0.0094
Range Fx T	<0.0001*	0.0375	0.0823	0.0240
Range Fx P	<0.0001*	0.05	0.0519	0.0122
STDEV Fx T	<0.0001*	0.05	0.1255	0.05
STDEV Fx P	<0.0001*	0.05	0.0823	0.0375

Fifty strides of a non-ataxic control group as well as 50 strides of an ataxic study group were analyzed and compared. T, thoracic limbs; P, pelvic limbs; *N*, number of steps; STDEV, standard deviation; CV, coefficient of variation; Highlighted cells, absolute values (restricted comparability); see Table 1 for detailed definitions of spatio-temporal and kinetic gait parameters and Supplementary Table S2 for descriptive statistics.

#### 3.3.2. Coefficients of variation of kinetic gait parameters

Table 3 shows higher CVs in dogs with SCI than in controls regarding IFz of both limb pairs, as well as PFy of the thoracic limbs and MFz and PFx of the pelvic limbs.

## 4. Discussion

In the current study, objective gait analysis in dogs with SCI suggestive of ataxia was performed and compared to a healthy control group to find differences in gait parameters and their CVs. Treadmill gait analysis with four built-in ground reaction force plates revealed increased spatio-temporal and kinetic variability, a shift in dispersion of stride phases and distinct alterations in ground reaction forces of the thoracic and pelvic limbs. Results of this study are consistent with those of previous studies quantifying gait abnormalities secondary to thoracolumbar SCI. In addition, we demonstrated that ataxia can be quantified in the pelvic as well as in the thoracic limb pairs using a computer-and video-assisted four-belt treadmill system and analysis of CV. Contrary to what could be expected, failure to place one foot per treadmill belt occurred in less than a handful of measurements, making data collection very manageable.

In 2001, Olby and colleagues identified the need for a reliable ataxia scoring system to improve assessment of the different therapies for acute SCI (35). Former experimental models used histologic (36–39) or immunhistochemical (40) evaluation of the damaged spinal cord to ensure objective assessment. However, in a clinical setting indirect methods for investigating functional outcomes of different therapies of SCI have been used (41–46). Treadmill gait analysis provides an additional useful tool to quantify the neurological assessment (20–22), providing more objective data for diagnosis and monitoring (35).

The ability to walk is a result of well-adjusted interactions between afferent and efferent components such as the cortex, the thalamus, the brain stem, as well as spinal locomotion centres, fine-tuned by cerebellar activity. Stimulation of this system results in an alternation of extension and flexion of the limbs and thus alternation of swing and support phase (1). Proprioceptive disturbances as well as paresis can lead to gait abnormalities. Usually, proprioceptive mechanisms are involved in self-regulation of body postures and motion by creating an awareness of the position of the limbs in space (1). Proprioceptive deficits result in incoordinated paw placement that is not necessarily observable in every single step cycle proceeded, demanding for more than the ten usually recorded cycles in orthopaedic studies. Whereas “pure” ataxia can be exclusively found in impairments of cerebellar or vestibular origin and can artificially be induced by drugs such as phenobarbital (PB) (11, 47). Ataxia in most patients with SCI are accompanied by paresis. In SCI, damage of the efferent tract in the ventral area of the spinal cord can cause paresis or plegia, whereas damage of the afferent tract in the dorsal area of the spinal cord are more likely to affect coordination causing ataxia by decreasing proprioceptive capabilities. Hypermetric movements, muscle atrophy, conscious avoidance of falling by not taking steps, as well as uncontrolled falling of the paw to the ground caused dogs with SCI to show objectively measurable differences in pelvic gait characteristics compared to controls. In contrast to PB-treated dogs of a similar study (11), not only ataxia but also paresis is likely to have contributed to severity of findings in the pelvic limbs in the current study.

We decided to use CV to analyse variability between groups. CV is a descriptive parameter for quantification of data spread, as in this study the intraindividual variability of a gait parameter and is therefore independent of factors such as body size and weight. Relative comparison of interindividual measurements on the treadmill are hereby possible. The higher CVs, the higher is variability of data and thus the level of ataxia. CVs have been successfully used before to quantify the variability of individual gait characteristics (31) in dogs (13, 48) and horses (14). In this study, CVs of spatio-temporal and kinetic gait parameters were increased not only in the pelvic limb pairs of the study group but also some CVs of the thoracic limbs were affected which was not expected by the authors prior to data analysis. Even though variability was more severe in the pelvic limbs, the authors did not expect CVs of the thoracic limbs to be significantly increased. Previous studies were based on the assumption that increased CVs are a potential sign of ataxia. In dogs with thoracolumbar SCI the thoracic limb pair is generally recognized as non-ataxic in the neurological examination. Other gait analysis studies using CVs to quantify ataxia in dogs with thoracolumbar myelopathy focused on the changes in variation (represented by parameters’ CVs) seen in the pelvic limbs, either because they only investigated the pelvic limbs (49), or because significant changes were only identified in the pelvic limbs (31). One study did identify thoracic limb CV changes, but they were not discussed (50).

Explaining increased CVs in the thoracic limbs of dogs with thoracolumbar SCI seems more controversial. Findings might either be suggestive of the role of the thoracic limbs for compensation, indicating that an increase in CV is not exclusive for ataxia but also includes potentially the detection of compensatory gait patterns. It is possible that the thoracic limb placements might only be compensatory, the human eye might not see the subtle compensatory mechanisms of the thoracic limbs to balance the ataxic and paretic pelvic limbs.

Another explanation might be the existence of a sensory system that does not only affect caudal regions of the damaged spinal cord but can moreover induce modifications in the coordination of the thoracic limbs as well. The thoracic and pelvic limbs pattern generators are distinct, but are coordinated during physiological gait (51). In simple terms, the rhythms of gait in the thoracic and pelvic limbs are generally of similar frequencies. Olby and colleagues have used this when scoring the severity of T3/L3 myelopathies (18). In dogs with a T3/L3 myelopathy, the pelvic limb gait frequency is different and usually lower than in the thoracic limbs. As the myelopathy improves the frequency of pelvic and thoracic limbs become similar again, with the lumbar central pattern generators (CPGs) providing input to thoracic CPGs and not vice versa (51). Alongside the spinal cord between the cervical and the lumbar CPGs rhythmogenic spinal circuits exist to co-ordinate the gait via ascending and descending, parallel and diagonal propriospinal cord tracts. These pathways play an important role for the task-and phase-dependent modulation of interlimb reflexes, the arrangement of muscle-specific contractions and the pelvic-thoracic limb coordination during locomotion in dogs and cats (51). Thus, an irritation of these pathways and the rhythmogenic spinal circuits could explain that also the thoracic limbs might be affected by certain ambulatory T3/L3 myelopathies.

Absolute measures were also compared in this study, with the foresight that differences seen might be mainly secondary to shape and size of the animal. Different limb sizes of dogs included in the study can partly explain differences seen in the absolute values between groups. Dogs with shorter legs take smaller steps, not related to their abilities in locomotion (52). Thus, relative values are more valid indicators for better comparability between groups. The majority of values in the current study were set in relation to the individual’s body weight or stride cycle or were illustrated as indices. In addition to that, for all gait parameter values, instead of simply comparing one mean per individual and parameter, data from 50 steps of each participant’s ipsilateral paw (total of 100 steps per individual limb pair) were compared. Gait aberrations in the pelvic limbs can partly be explained by the influence of paresis and the rest by ataxia, creating the need for compensatory mechanisms in the thoracic limbs in order to keep the dog capable of locomotion.

Compensatory mechanisms such as the shift from single to double support and elongation of stance duration were longer in dogs with idiopathic epilepsy treated with PB as shown in the current study (11). In this study, however, dogs with SCI had higher CVs than PB-treated dogs. One possible explanation is that dogs with PB-induced ataxia had the ability of applying more sufficient and controlled compensational techniques or that the level of ataxia was more subtle. SCI causes not only ataxia but also motor deficits could hinder the SCI-ataxic dog from implementing compensatory mechanisms, making them appear more involuntary and less effective. The degree of gait aberrations in the pelvic limbs appeared to be more severe in dogs with spinal ataxia than in dogs under anti-seizure treatment. In addition, four of five participating ataxic dogs with IVDD in this study underwent hemilaminectomy. Previous studies have already indicated a lasting effect on stance time of the ipsilateral as well as the contralateral limb in Dachshunds even after surgical decompression in comparison to normal dogs (53).

### 4.1. Study limitations

In order to guarantee animal welfare in accordance with the Lower Saxony State Office for Consumer Protection and Food Safety in Oldenburg, Germany number of participating dogs in this animal experiment was to be officially approved and thus limited. A control group was approved for clinic-owned beagles only, limiting the diversity of breeds in controls, as well as the overall amount of dogs in both groups. Nevertheless, several significant findings were observed, after corrections were applied. Considering the result of this study, further studies are needed with more diverse dog populations and SCI status to demonstrate repeatability of study outcomes and identify differences. The treadmill system used in this trial is based on specialised equipment with high-sensitive sensors. In addition, further studies could aim to identify potential correlations between the results of this study setting and those of validated ordinal open field walking scales.

Results for craniocaudal (Fy) and mediolateral (Fx) GRF might be less valid than for Fz since stride events “paw strike” and “paw off” were set mainly according to start and end of detection of vertical ground reaction forces (Fz) of kinetic gait analysis. During gait analysis, the treadmill reflective body markers did fall off of dogs’ paws sometimes, causing the gait analysis software to struggle detecting and calculating spatial gait parameters. The following data sets “stride and step time” of the group of dogs showing spinal ataxia were missing: One thoracic limb data set, one pelvic limb data set, one thoracic and pelvic limb data set. For future studies an additional objective quantification of distances between thoracic as well as pelvic paws, summarised as “Base of Support” (50), inclusion of kinematic analysis with electromyographic activity detection and selection of larger sample sizes with higher diversity of participating canines and different neuroanatomical localizations of SCI would be useful. If the software system used for data selection in this study will be supplemented for further investigations, the order of paw placements could also be a gait parameter complementing existing data sets. Consecutively, paw placement orders might reveal an additional aspect of prevalent patterns of compensatory mechanisms in dogs with SCI.

## 5. Conclusion

This study was the first to perform objective gait analysis of dogs with thoracolumbar SCI on a treadmill with four built-in ground reaction force plates with a focus on CVs of the gait parameters measured. We were able to show that variability in spatio-temporal and kinetic gait parameters were increased, stride phases were shifted and GRF were altered. Differences were found not only in the pelvic, but also in the thoracic limb pairs. Although variability of pelvic limbs was affected to a greater extent than thoracic limbs, findings imply that increased CVs are a tool for detecting not only ataxic but also compensatory gait patterns, or that we have to rethink our understanding of SCI and its potential influence on neurological segments cranial of the damaged T3-L3 spinal cord segments. In contrast to PB-treated ataxic dogs with IE in a similar previous study (11), gait parameters and their variability were more affected and compensatory mechanisms seem less sufficient in dogs with SCI. Treadmill analysis was well tolerated and not invasive nor distressful for participating dogs and could complement gait analysis systems using body-worn sensors and machine learning techniques (54). Gait analysis as performed in this study could be an alternative tool for follow-up monitoring studies on long-term functional outcome of dogs with SCI, before and after neurosurgical intervention.

## Data availability statement

The raw data supporting the conclusions of this article will be made available by the authors, without undue reservation.

## Ethics statement

The animal studies were approved by Lower Saxony State Office for Consumer Protection and Food Safety (LAVES), reference number for this project: 20A555. The studies were conducted in accordance with the local legislation and institutional requirements. Written informed consent was obtained from the owners for the participation of their animals in this study.

## Author contributions

TS, FT, SM, and HV performed data acquisition and planned the experiment(s). TS and AM-A ran the experiment. TS analyzed the data. TS, FT, SM, AM-A, and HV participated in manuscript writing. All authors contributed to the article and approved the submitted version.

## Funding

The research project was fund and equipment were provided by the Department of Small Animal Medicine and Surgery of the University of Veterinary Medicine Hannover, Germany.

## Conflict of interest

The authors declare that the research was conducted in the absence of any commercial or financial relationships that could be construed as a potential conflict of interest.

## Publisher’s note

All claims expressed in this article are solely those of the authors and do not necessarily represent those of their affiliated organizations, or those of the publisher, the editors and the reviewers. Any product that may be evaluated in this article, or claim that may be made by its manufacturer, is not guaranteed or endorsed by the publisher.
