# Supplementary material for: Quantification of spinal ataxia in dogs with thoracolumbar spinal cord injury
Source: Front Vet Sci. 2023 Aug 8;10:1183755. doi: 10.3389/fvets.2023.1183755 (PMC10442642; doi:10.3389/fvets.2023.1183755)
Supplement: Supplementary file 1 [file Data_Sheet_1.PDF]

Supplementary Material

Quantification of spinal ataxia in dogs with thoracolumbar spinal cord injury

Tamara Sherif[1], Friederike Twele[1], Sebastian Meller[1], Alexandra Müller-Anders[1], Holger A. Volk[1*]

[1] Department of Small Animal Medicine and Surgery, University of Veterinary Medicine Hannover, D-30559 Hannover, Germany

*** Correspondence:**
Holger A. Volk
holger.volk@tiho-hannover.de

1 Supplementary Tables

Supplementary Table 1. Descriptive statistical analysis of parameter values and coefficients of variation (* p <= 0.05, Mann-Whitney test, FDR Q = 0.05) of spatio-temporal gait parameters. 50 strides of a non-ataxic control group as well as 50 strides of an ataxic study group were analyzed and compared. T = thoracic limbs; P = pelvic limbs; N = number of steps; CV = Coefficient of variation; highlighted cells = absolute values (restricted comparability); See **Table 1** for detailed definitions of spatio-temporal gait parameters.

SPATIO-TEMPORAL	**Control group** (n=6, N=600)				**Study group** (n=5, N=500)			
	**Median**	**Range**	**Variation**	**CV**	**Median**	**Range**	**Variation**	**CV**
**Stride length T**	447.7	353.0-546.9	1756.71	9.40	319.3	103.8-475.1	10664.46	33.71
**Stride length P**	480.4	351.1-572.4	1785.78	8.84	304.6	183.3-487.9	5733.17	22.32
**Step length T**	243.3	186.0-310.1	544.27	9.56	176.2	49.4-259.8	3132.10	33.04
**Step length P**	245.9	168.2-322.4	820.88	11.61	169.4	17.6-299.5	3634.25	34.71
**Stride time T**	0.76	0.57-1.05	<0.01	9.57	0.68	0.38-1.12	0.02	20.76
**Stride time P**	0,76	0.61-1.04	<0.01	9.39	0.81	0.35-1.39	0.03	21.49
**Step time T**	0.38	0.27-0.63	<0.01	11.12	0.34	0.17-0.75	<0.01	23.99
**Step time P**	0.38	0.29-0.63	<0.01	10.69	0.41	0.20-0.92	0.01	28.00
**Stance time T**	0.51	0.27-0.91	<0.01	11.73	0.47	0.07-0.97	0.02	25.08
**Stance time P**	0.47	0.34-0.91	<0.01	10.94	0.52	0.19-0.99	0.01	21.01
**Swing time T**	0.25	0.17-0.54	<0.01	12.86	0.20	0.08-0.52	<0.01	24.24
**Swing time P**	0.29	0.16-0.43	<0.01	13.21	0.30	0.00-0.78	0.01	37.26
**rel. Step length T**	54.8	45.2-65.8	10.93	6.03	55.2	33.8-84.0	43.75	11.86
**rel. Step length P**	51.5	39.4-79.5	16.07	7.76	50.6	8.9-110.1	234.14	29.89
**rel. Step time T**	50.0	40.0-62.4	7.89	5.62	50.0	27.4-134.2	44.35	13.20
**rel. Step time P**	50.0	39.4-64.3	6.11	4.94	50.0	19.1-151.3	87.67	18.57
**Single support T**	33.3	23.1-51.4	9.19	9.05	29.6	12.3-45.3	23.53	16.37
**Single support P**	38.9	23.2-45.8	10.31	8.35	34.6	16.0-58.7	51.98	20.83
**Double support T**	33.3	6.7-44.4	18.68	13.09	40.0	17.8-74.5	61.10	19.31
**Double support P**	22.2	14.5-41.8	18.71	18.76	29.8	5.7-58.1	119.68	35.93
**Swing phase T**	33.7	24.1-53.5	9.99	9.44	30.0	12.2-46.8	25.79	17.02
**Swing phase P**	38.9	25.0-45.1	9.81	8.14	35.1	17.1-63.4	55.94	21.11

Supplementary Table 2. Descriptive statistical analysis of parameter values and coefficients of variation (* p <= 0.05, Mann-Whitney test, FDR Q = 0.05) and kinetic gait parameters. 50 strides of a non-ataxic control group as well as 50 strides of an ataxic study group were analyzed and compared. T = thoracic limbs; P = pelvic limbs; N = number of steps; STDEV = standard deviation; CV = Coefficient of variation; highlighted cells = absolute values (restricted comparability); See **Table 1** for detailed definitions of kinetic gait parameters.

KINETIC	**Control group** (n=6, N=600)				**Study group** (n=5, N=500)			
	**Median**	**Range**	**Variation**	**CV**	**Median**	**Range**	**Variation**	**CV**
**PFz T**	59.4	45.7-72.6	12.79	6.02	54.0	28.8-96.7	52.48	13.18
**PFz P**	35.2	26.1-51.6	18.08	11.92	39.6	13.8-69.0	57.22	18.88
**MFz T**	40.9	28.4-48.0	7.60	6.79	36.1	13.8-48.6	24.75	13.76
**MFz P**	25.1	15.8-33.7	3.95	7.87	25.6	1.5-42.7	25.44	19.86
**PFy T**	13.2	6.0-30.8	8.99	22.14	9.3	0.0-24.8	32.45	58.35
**PFy P**	5.9	0.0-16.5	13.44	61.18	4.4	0.0-20.2	13.39	81.13
**PFx T**	6.6	0.0-28.4	28.56	79.71	6.9	0.0-27.6	48.99	88.01
**PFx P**	6.4	0.0-16.8	26.45	79.24	5.3	0.0-21.7	12.41	60.67
**IFz T**	20.7	9.8-36.4	7.48	13.16	17.5	2.1-33.8	16.91	23.13
**IFz P**	11.9	6.3-22.1	2.52	13.30	13.0	0.9-29.0	18.31	31.56
**Range Fz T**	86.4	58.5-119.0	101.58	11.59	56.8	23.5-105.4	355.44	29.66
**Range Fz P**	51.9	22.3-84.6	81.04	17.30	41.1	13.7-77.9	189.69	30.08
**STDEV Fz T**	23.3	17.2-35.1	9.82	13.24	14.7	6.2-28.9	34.27	35.31
**STDEV Fz P**	11.3	5.2-21.5	6.85	22.34	10.0	0.0-20.8	12.85	32.22
**Range Fy T**	27.1	12.3-57.9	28.72	19.56	15.6	6.6-39.3	40.89	37.32
**Range Fy P**	23.0	11.0-47.8	42.12	26.98	18.3	0.0-48.8	31.58	29.30
**STDEV Fy T**	23.0	11.0-47.8	2.18	18.28	18.3	0.0-48.8	3.70	44.25
**STDEV Fy P**	5.8	3.2-11.0	2.22	24.46	4.5	0.0-8.9	1.36	25.96
**Range Fx T**	19.0	11.0-46.6	11.78	17.67	16.6	6.1-40.7	37.54	34.63
**Range Fx P**	17.6	7.2-39.3	47.38	37.33	10.8	0.0-40.7	46.03	52.21
**STDEV Fx T**	4.9	3.0-13.4	1.30	21.91	4.1	1.5-10.5	3.35	40.28
**STDEV Fx P**	3.8	1.7-9.5	4.03	45.33	2.5	0.0-8.8	2.40	51.81

Supplementary Table 3. Statistical analysis of symmetry indices (* p <= 0.05, Mann-Whitney test, FDR Q = 0.05). 50 strides of a non-ataxic control group as well as 50 strides of an ataxic study group were analyzed and compared. T = thoracic limbs; P = pelvic limbs; See **Table 1** for detailed definitions.

SYMMETRY INDICES	**Parameter values**	
	**P value**	**FDR threshold**
**Symmetry Index T**	0.3290	0.05
**Symmetry Index P**	0.0823	0.0240
